# Assessment of Anxiety in Cognitive-Behavior Therapy in Young University Students with Autism Spectrum Disorders: A Review

**DOI:** 10.3390/ejihpe11040111

**Published:** 2021-12-02

**Authors:** Sarah Sánchez-Cueva, Yurena Alonso-Esteban, Francisco Alcantud-Marín

**Affiliations:** Department of Developmental and Educational Psychology, University of Valencia, 46010 Valencia, Spain; masancue@alumni.uv.es (S.S.-C.); Yurena.Alonso@uv.es (Y.A.-E.)

**Keywords:** autism spectrum disorder, anxiety, cognitive-behavioral therapy, university students, systematic review

## Abstract

The university provides academic support to disabled students, however, few institutions provide academic, extra-academic or preventive support to students with Autism Spectrum Disorders High-Functioning (ASD-HF). Among the most commonly requested needs is support for coping with anxiety arising from academic and social activity itself. When planning an intervention program, it is necessary to screen those who have problems and are likely to benefit from such a program. In this article we propose a systematic search for measures of anxiety for young people and adults with ASD-HF. Of a total of 683 documents, only 7 met the selection criteria. Of these, a total of 35 tools were detected, of which only 11 mediated anxiety. Screening should be carried out for all students, so that we can detect the “risk” of anxiety disturbance in all cases and, in particular, in students with ASD-HF. However, the instruments eligible for assessing intervention outcomes should be agreed upon in order to be able to compare results from different trials.

## 1. Introduction

Autism Spectrum Disorders (ASD) are one the most common neurodevelopmental disorders, characterized by deficits in communication and social interaction and restricted and repetitive behaviours [1]. Recent epidemiological data suggested an estimated prevalence (1 to 2%) of the young [2,3] and adult populations [4]. Among the reasons given to explain the increased prevalence is an increase in the number of cases of high-functioning autism spectrum disorder (ASD-HF) [5]. In other words, it has been suggested that the increase in overall prevalence is likely due to a greater ability to recognize children without intellectual disability, with milder forms of ASD (including high-functioning autism spectrum disorder and Asperger’s syndrome) [6]. Additionally, although ASD is first evident at an early age, identification and diagnosis of ASD can be delayed and some individuals may remain undiagnosed until they reach a later age [7,8]. In fact, many of today’s adults with ASD-HF were not diagnosed as being on the autism spectrum when they were children [9]. 

Research and practice to date has primarily focused on children [10] in front of the research on adults, which has not been as extensive as that on children. However, some small-scale studies have contributed to a further understanding of the postsecondary education experiences of individuals with ASD [11]. 

A growing number of students with ASD are enrolling in higher education. Recent research has estimated that 1% of individuals diagnosed with ASD prior to starting school continue to higher education [12]. Existing research suggests that these people will continue to exhibit interpersonal deficits throughout their adulthood [13]. Research suggests that individuals with ASD in particular suffer from a lack of post-high school supports [14]. In addition, some students may not yet be diagnosed when they start university, and others do not declare their status or access academic support services. White et al. [15] analyzed the university population using the Ritvo scale (Ritvo Autism Asperger Diagnostic Scale-Revised: Ritvo et al. [16]), concluding that “between 0.7 and 1.9% of university students may meet the diagnostic criteria for ASD” ([15], p. 683). Thus, these individuals have missed out on the benefits that educational and therapeutic programs designed to meet their needs would have brought them, and are therefore at greater risk of having academical, personal or social problems.

Despite awareness of the growing number of college-bound youth with ASD, there is relatively little information available about the unique needs of this group. As far as understanding the experiences of young adults with ASD is concerned, the field continues to be marked by a lack of studies in the post-secondary education domain. Many young adults with ASD face an important stage in their development when they enter university and transition to adulthood [17]. Clinical and counseling researchers have identified a range of potential challenges for university students on the autism spectrum [18].

While some young adults with ASD have the cognitive ability to meet the educational demands of university, the inherent characteristics of ASD may affect their university experience, making them face numerous academic, social and independent living challenges [17]. In fact, the number of university students with ASD who go on to graduate is very low [12]. The reasons for drop-out are essentially a lack of specialized support services [13].

Therefore, students with ASD-HF have the intellectual competences to be able to achieve a degree, however, they are considered “population at risk” due to the conditions of the disorder itself. [19]. The reason for this condition is that increasing or varying the demands of social interaction may increase the core symptoms of ASD or other comorbid disorders may appear [20] including anxiety disorders, depression, and suicidal thoughts and behaviors [21]. Executive dysfunction, emotional dysregulation, cognitive rigidity, social deficits, etc., may be behind academic failure that is not justified either by the student’s intellectual ability or motivation. As a consequence of these or other disturbances and in combination with the stress caused by university life, university students with HF-ASD, such as the general population with ASD, experience anxiety crises [22] and other mental disorders [23]. Gurbuz et al. [24] conducted a study comparing a group of students with and without HF/ASD, concluding that 54% of students with ASD-HF reported some mental disorder (depression or anxiety) compared to only 17% of neurotypical students. 

The non-pharmacological treatment with the most documented efficacy for Anxiety Disorders is undoubtedly the cognitive-behavioral therapeutic approach [25] and also in the ASD population [26,27]. There is a large body of literature on the use of CBT in children and young people with ASD demonstrating its effect as a treatment for anxiety and depression. [28,29,30,31] Even though anxiety disorders are so prevalent, support or treatment of anxiety disorders among students with HF/ASD is not addressed as such in university support programs [23]. 

Providing efficient psychological and academic support to university students with ASD-FH is a challenge for the whole university community and must respond to the specific needs of this group. Anderson, Carter and Stephenson [32] developed a survey to explore the experience of students at eight universities in Australia which, among other things, showed that the most frequent comorbid disorder was anxiety (65.2% of respondents). When asked about services and level of satisfaction, it is at least curious that 85.4% never used stress management training services and those who did found it not at all or not very useful.

Consequently, the development of effective cognitive-behavioral treatment (CBT) programmed to train students with ASD-HF in effective coping techniques for anxiety, focused on the characteristics and needs of university students and that are available to them throughout their time at university, is particularly needed.

In this sense, we proposed the design of a cognitive-behavioral intervention programmed for anxiety (PICCA, Programa de Intervención Cognitivo-Conductual en Ansiedad) to improve coping with anxiety in university students with ASD-HF, and in order to gather adequate models, both of the contents themselves and of the measures to determine their effectiveness and acceptance, we planned a documental review. This paper aims to identify and review current best practices for anxiety assessment in clinical trials of cognitive-behavioral therapy (CBT) for young adults with ASD-HF.

## 2. Methods

**Figure 1:**
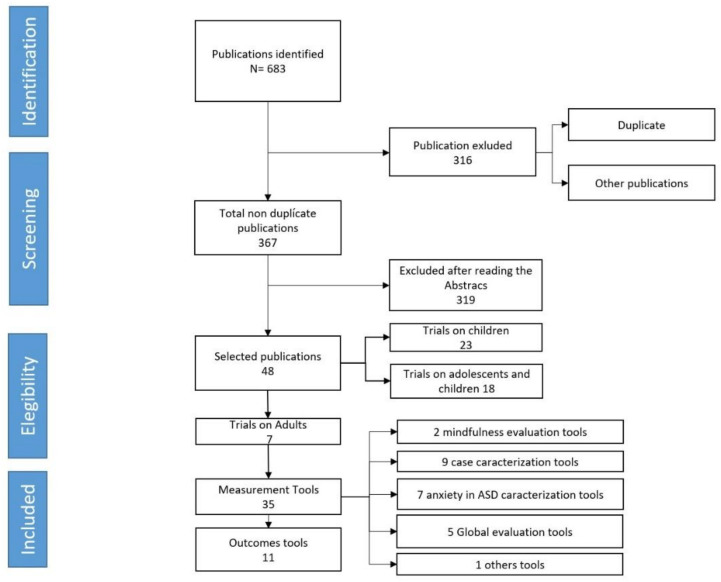
Flow of the search process.

The criteria for the search, selection and evaluation of documents have been carried out according to the PRISMA recommendations [33] (See flow in Figure 1).

### 2.1. Eligibility Criteria and Study Selection

We searched three databases: ProQuest-PsychArticles, ProQuest-ERIC and PubMed. The search was narrowed down to 2015 to 2020. Several tests were performed with different search terms (“CBT” AND “Anxiety” AND “Autism”). These terms must appear in the title or abstract of the indexed document. The search was performed by accessing all databases with the online search interface TROBES of the Documentation and Library Service of the “Universitat de València” (Valencia, Spain). The search ended on 30 January 2021, although in order to keep the list as up to date as possible, a Google alert was maintained with the same search terms.

### 2.2. Inclusion Criteria

The review was limited to studies published in double-blind peer-reviewed scientific journals. Only studies reporting results with at least one measure of anxiety with psychometric instruments and where the age of application was within the range of 17 and 25 years were selected.

### 2.3. Exclusion Criteria

Studies were excluded if any of the following criteria were met: (a) systematic literature review and meta-analysis studies; (b) articles focusing on the assessment of anxiety by biological or physiological means; and (c) clinical trials developed on people without an ASD diagnosis or outside the age range were also excluded.

### 2.4. Procedure

A total of 683 documents were initially identified. The documents were reviewed, firstly excluding those articles that were duplicated in the different databases consulted. Secondly, the abstract of each document was consulted to exclude publications that did not meet the inclusion criteria, selecting a total of 48 documents. Identification and selection were conducted by S.S.C. and Y.A.E. independently, and discrepancies were solved by F.A.M.

## 3. Results

Of the 48 papers selected, 23 studies refer to clinical trials in children (up to the age of 11 years), 18 trials in adolescents (12–16 years) and only 7 in adults (17 years and older). We did not find any CBT trials for anxiety on a sample of university students with ASD. Consequently, we will take the seven studies developed for adults with ASD-HF as a reference. As shown in Supplementary Materials Table S1, the seven studies collected present some methodological diversity following to some extent the results of previous reviews [34]. The 46 measurement instruments used can be classified into 3 main groups: (a) characteristics of the sample; (b) relationship between ASD symptoms and anxiety; and (c) outcome measures. 

### 3.1. Subsection Instruments for Assessing Core ASD Symptoms 

There is usually a great deal of overlap in the case definition in the inclusion criteria so that the instruments used are the “gold standard” for diagnosis, such as ADOS (or the current version ADOS-2) [35], ADI-R [36], AQ [37], SRS [38] and the SCQ [39] for the diagnostic characterization of ASD, ADIS-5 [40] for the diagnosis of Anxiety Disorders and the Wechsler Scale (WAIS-IV; [41]) for determining IQ. 

### 3.2. Clinical Assessment of Anxiety in Young Adults with ASD 

There are several studies that attempt to construct an explanatory model of the relationship between ASD and Anxiety Disorders. Some authors [42] explain some symptoms of people with ASD, such as the need for environmental invariance, restricted interests or rigidity, as a response to the anxiety produced by the social world. Other authors [43] put forward anxiety as a reaction to the difficulties of understanding what is happening and to the feeling of uncertainty and permanent helplessness in people with autism. Wood and Gadow [44] have developed a hypothetical explanatory model of the appearance and maintenance of anxiety disorders in people with ASD based on the limitations of people with ASD (social confusion, the unpredictability of relationships and social encounters, social rejection and victimization, helplessness, negative experience by punishments or restrictions by repetitive and stereotyped behaviors or aversive sensory experiences) can act as stressors in people with ASD and, as a consequence, cause an alteration in the mood and increase anxiety (in an acute or continuous way). As a consequence, there is some discrepancy between studies. Firstly, some trials do not use these tools, while others try to obtain data to corroborate one relationship or the other. In this sense, in our search we found instruments to assess irrational thoughts such as IBI [45], rumination RRQ [46], intolerance to uncertainty IUS-12 [47] and other disorders such as obsessive thoughts Y-BOCS [48], depression GMS [49] or alexithymia BVAQ-ID [50], 2001) and TAS-20 [51].

### 3.3. Outcome Measures in CBT Intervention Trials

There is also a diversity of criteria for assessing the outcomes of the intervention. On the one hand, there are measures related to global health status such as CORE-OM [52], EQ-5D [53], levels of severity of the disorder CGI-S [54] and quality of life WHOQOL-BREF [55]. Studies using mindfulness strategies also use specific assessment tools to evaluate changes in this construct such as FFMQ [56] and MAAS [57].

On the other hand, the direct measures of anxiety that will be studied in more detail are listed in Table 1. Among the 11 selected anxiety measures, we will eliminate the description of three of them addressed to parents (RCADS-P [58], CASI-P [59], FAS-A [60]). 

The BASC-2 system [61] assesses the emotional, behavioral and adaptive functioning of adolescents aged 12 to 21 years. The subscales address a wide range of both internalizing and externalizing concerns. It consists of five questionnaires: (1) Teacher Rating Scale (TRS), (2) Parent Rating Scale (PRS), (3) Self-Report Personality (SRP), (4) Structured Developmental History (SDH) and (5) Student Observation System (SOS). The measures included in the TRS and PRS questionnaires are anger coping, bullying, social relationship disorders, self-control, executive function, negative emotionality and resilience. The PRS has three age-specific versions (children 8–11 years, adolescents 12–21 and young adults 18–25 years) and measures school problems, internalizing problems, inattention/hyperactivity, personal adjustment and a global composite score, the Emotional Symptoms Index (ESI). The SDH is a form used to collect social, psychological, developmental, educational and medical information about the person being assessed. It can be completed by the clinician during an interview with the parent or guardian or can be completed as a questionnaire by the parent. The SOS is a direct observation procedure, using the technique of temporal sampling to record both positive and negative behaviors.

GAD 7 [62] is a seven-item questionnaire measuring generalized anxiety that is used for screening purposes. Psychometric data provide good reliability, construct validity and excellent sensitivity (0.89) and specificity (0.82) values. LSAS [63] is a 24-item questionnaire that lists general social situations in which anxiety generation and avoidance drive are asked to be assessed on a four-point Likert scale. There are two administration formats, the clinician-administered version (Clinician-administered version LSAS-CA) and the self-reported version (LSAS-SR). STAI [64] is a classic questionnaire in the assessment of anxiety with several editions in existence. It comprises two separate scales assessing state and trait anxiety, each with 20 items. The questionnaire has good construct validity, internal consistency (0.797 for anxiety/state and 0.781 for anxiety/trait) and test–retest reliability 0.850 [69]. 

The Hospital Anxiety and Depression Scale (HADS; [66]) was designed to measure general mood and anxiety symptoms in non-psychiatric inpatients. It is divided into two subscales: seven items related anxiety (HADS-A) and another seven items to depression (HADS-D). The reliability of both scales is around 0.78–0.93. [70].

The ASA-A scale (Anxiety Scale for Autism-Adults) [67] is a 20-item self-report screening questionnaire divided into three subscales: Social Phobia (SP), Anxiety (AA) and Uncertainty (U). ASA-A has adequate internal consistency (0.89–0.83) and test–retest reliability (0.82) and cross-validity with HADS (0.47–0.69). The Hamilton Anxiety Scale (HAM-A) is one of the first scales for the assessment of anxiety symptoms where the clinician assesses anxiety symptoms and severity, severity and impairment in adults over the past week [68].

In summary, the specific measures used are mostly self-report instruments, sometimes supplemented by questionnaires reported by parents or caregivers. With the exception of the ASA-A scale, there are no specific instruments to measure anxiety in adults with ASD, and we have not found any that assess the stressors inherent to university contexts and environments.

## 4. Discussion

Despite the growing awareness of the increasing population of young adults diagnosed with an ASD-HF who are in higher education [15,71] and the need for academic and psychological supports [13], as a result of the search, we can conclude that very little research is available to help understand the emotional problems in this population group. Although many university institutions offer academic support for students with ASD, only a few offer extracurricular support or prevention programming [24,72,73,74]. Indeed, the first conclusion of this review is the lack of studies on the use of CBT in university students with HF-ASD. There is a clear need to develop CBT programs tailored to the anxiety problems of this population. The few studies found in our review report a paucity of assessment instruments suitable for the detection and monitoring of anxiety-related problems in young adults with ASD-HF and, by extension, in university students.

Currently, and as a consequence of an increased presence of university students with ASD-HF, the literature points to an increased knowledge, awareness and acceptance of people with ASD on university campuses [75,76,77].

However, while there is much literature on diagnosis and psychoeducational intervention in autistic children, research in youth and adults is scarcer [78]. In this study, we have focused on screening instruments to detect and monitor the evolution of university students with ASD-HF who attend a training program to improve coping in stressful academic situations.

It should be noted that the tools used in the seven selected clinical trials are mostly clinical tools. Given the idiosyncrasies of people with ASD-HF, it is quite possible that tools administered in the form of a clinical interview should be used to ensure that each of the questions asked is understood and identified [79]. In this sense, it is necessary to have instruments such as LSAS with clinically administered and self-reported versions and additionally, multi-reporters with versions for parents and caregivers. Convergence rates between different informants can improve the assessment of anxiety disorders and changes throughout the intervention program. 

One of the weaknesses, as pointed out by Provenzanil et al. [80], is the lack of uniformity in the selection of one or more instruments to measure the results of clinical trials that would allow comparison of one method with another. Similarly, the use of self-reports in individuals with ASD has been discussed, and it has even been reported that self-reports in individuals with ASD have been used to measure the results of clinical trials [80], and it has even been reported that the psychometric properties of some measures have not been adequately studied [81].

In short, this review has allowed us to determine a twofold need: On the one hand, the development of support and intervention program for university students with HF/ASD to improve coping with anxiety and other associated mental health problems. On the other hand, and closely linked, there is a need for the development of measurement instruments adapted in two directions, firstly, with respect to stressors related to university life and secondly, adapted to be used by students with ASD-HF.

Limitations of this study

This review needs to be complemented by others that broaden the search spectrum in order to confirm the lack of CBT programs for anxiety in autistic young adults.

## Figures and Tables

**Table 1:** Description of the anxiety assessment tools used.

			No. of Items	Age	Reliability		
					Consistency	Stability	Interjudice
1. BASC-2	Behavior Assessment System for Children, Second Edition [61]			2 a 25	TRS 0.90 PRS 0.90 SRP 0.90	TRS 0.80 PRS 0.90 SRP 0.80	TRS 0.19 to 0.82 PRS 0.74 0.77
2. GAD-7	General Anxiety Disorder-7 [62]		7	18–95	0.92		0.83
3. LSAS	Liebowitz Social Anxiety Scale [63]		24	18–95	0.80–0.85		
4. STAI-T	State-Trait Anxiety Inventory for Adults [64]		20/20	Adults	0.97	0.54	
5. BAI	Beck Anxisety Inventory [65]		21	17–95	0.92	0.75	
6. HADS A&D	Hospital Anxiety and Depression Scale checklist [66]		14	18–95	0.87	0.74	
7. ASA-A	Anxiety Scale for Autism-Adult [67]		20	Adults	0.89	0.82	
8. HAM-A	Hamilton Anxiety Scale [68]		14	Adults	0.77–0.92		

## Supplementary Materials

The following are available online at https://www.mdpi.com/article/10.3390/ejihpe11040111/s1, Table S1: Description of the characteristics of the seven analyzed studies.
