# Supplementary material for: Assessment of Anxiety in Cognitive-Behavior Therapy in Young University Students with Autism Spectrum Disorders: A Review
Source: Eur J Investig Health Psychol Educ. 2021 Dec 2;11(4):1571–80. doi: 10.3390/ejihpe11040111 (PMC8699995; doi:10.3390/ejihpe11040111)
Supplement: Supplementary file 1 [file ejihpe-11-00111-s001.zip › ejihpe-1461021-supplementary.pdf]

Table S1: Description of the characteristics of the studies analyzed (N=7).

Study	Intervention model	Design	Duration		N	Age	Measures
			Programme	Sessions			
1. Ehrenreich-May, y otros, 2020	AFIYA Cognitive-Behavioral program based on BIACA and PEERS	Single Case Study with multiple baselines	16 weeks	90 minutes	6	15-21	1. ADIS-IV-C/P: Anxiety Disorders Interview Schedule for DSM-IV, Child Version, Child and Parent Report Forms (Silverman & Albano, 1996)<br>2. ADOS: Autism Diagnostic Observation Schedule) (Lord, y otros, 2012)<br>3. RCADS and RCADS-P: Revised Child Anxiety and Depression Scales, child and parent versions (Ebesutani, y otros, 2010)<br>4. BASC-2: Behavior Assessment System for Children, Second Edition (Reynolds & Kamphaus, 2004)<br>5. SRS: Social Responsiveness Scale (Constantino & Gruber, 2005)<br>6. SCQ: Social Communication Questionnaire (Rutter, Bailey , & Lord, 2003)
2. Gaigg, y otros, 2020	Online Cognitive Behavioral Therapy and Mindfulness	Randomized Clinical Trial			54	23-66	1. ADOS: Autism Diagnostic Observation Schedule) (Lord, y otros, 2012)<br>2. SRS: Social Responsiveness Scale (Constantino & Gruber, 2005)<br>3. AQ; Autism Spectrum Quotient (Baron-Cohen, Wheelwright, Skinner, Martin, & Clubley, 2001).<br>4. WAIS-4: Wechsler Adult Intelligence Scale (Wechsler, Wechsler Adult Intelligence Scale 4th ed, 2012)<br>5. GAD-7 (General Anxiety Disorder-7; (Spitzer, Kroenke, Williams, & Löwe, 2006),<br>6. LSAS (Liebowitz Social Anxiety Scale -; (Liebowitz, 1987),<br>7. STAI-T; (Spielberger, Gorusch, Lushene, Vagg, & Jacobs, 1983)<br>8. BAI (Beck & Steer, 1993)<br>9. HADS A&D Hospital Anxiety and Depression Scale checklist (Zigmond & Snaith, 1983)<br>10. CORE-OM Clinical Outcomes in Routine Evaluation - Outcome Measure (Evans, y otros, 2000)<br>11. BVAQ-ID Bermond-Vorst Alexithymia Questionnaire (Vorst & Bermond, 2001)<br>12. IUS-12 12-item Intolerance of Uncertainty Scale (Carleton, Norton, & Asmundson, 2007)<br>13. FFMQ Five-Faceted Mindfulness Questionnaire (Baer, Smith, Hopkins, Krietemeyer, & Toney, 2006)
3. Ordaz, Lewin, & Storch, 2018	TALAA Treatment for Anxiety in Late Adolescents With Autism	Single Case Study	16	60	1	18	1. ADIS-5 (Anxiety Disorders Interview Schedule DSM-5) (Brown & Barlow, 2013)<br>2. Y-BOCS; Yale-Brown Obsessive Compulsive Compulsive Scale Symptom Checklist (Goodman, y otros, 1989)

							3. CGI-S (Clinical Global Impression of Severity (Guy, 1976)<br>4. ADOS: Autism Diagnostic Observation Schedule) (Lord, y otros, 2012)<br>5. WASI-II; Wechsler Abbreviated Scale of Intelligence-Second Edition (Wechsler, 2011)<br>6. BAI (Beck & Steer, 1993)<br>7. CAIS-P; Child Anxiety Impact Scale-Parent (Langley, Bergman, McCracken, & Piacentini, 2004)<br>8. Family Accommodation Scale for Anxiety (FAS-A; (Lebowitz, y otros, 2013)
4. Parr, y otros, 2020	Personalised Anxiety Treatment-Autism (PAT-A©) with Mindfulness and VR Exposure	Randomized Clinical Trial with control group.	12 + individual sessions	undetermined	40	>18	1. SRS: Social Responsiveness Scale (Constantino & Gruber, 2005; 2012)<br>2. Anxiety Scale for Autism-Adult (ASA-A) (Rodgers, y otros, 2020):<br>3. HADS Hospital Anxiety and Depression Scale checklist (Zigmond & Snaith, 1983)<br>3. ADIS-5 (Anxiety Disorders Interview (Brown & Barlow, 2013)<br>4. WHOQOL-BREF (WHO Quality of Life-BREF) (The WHOQOL Group, 1998)<br>5. EQ-5D EuroQoL 5 dimensions, 5 levels health survey (Herdman, y otros, 2011)<br>6. TAS-20 Toronto Alexithymia Scale-20 (Bagdy, Parker, & Taylor, 1994)<br>7. W-ADL Waisman Activities of Daily Living Scale (Rodgers, y otros, 2017)<br>8. Reasonable adjustments. Ad hoc checklist based on (Lipinski, Blanke, Suenkel, & Dziobek, 2019)
5. Sizoo & Kuiper, 2017	Cognitive Behavioral Therapy and Mindfulness (MBSR)	Two-group experimental trial	13	90	59	>18	1. HADS Hospital Anxiety and Depression Scale checklist (Zigmond & Snaith, 1983).<br>2. GMS (Global Mood Scale) (Denollet, 1993)<br>3. RRQ (Rumination-Reflection questionnaire) (Trapnell & Campbell, 1999)<br>4. IBI (Irrational Beliefs Inventory) (Koopmans, Sanderman, Timmerman, & Emmelkamp, 1994)<br>5. SRS Social Responsiveness Scale (Constantino & Gruber, 2005)<br>6. MAAS (Mindful Attention Awareness Scale) (Brown & Ryan, 2003)<br>7. ADOS (Autism Diagnostic Observation Schedule) (Lord, y otros, 2012)<br>8. ADI-R (Autism Diagnotic Interview- Revised (Lord, Rutter, & Le Couteur, 1994)
			11	120	18	22-48	1. LSAS Liebowitz Social Anxiety Scale LSAS (Liebowitz, 1987)

6. Spain, Blainey, & Vaillancourt, 2017	Group Cognitive Behavioral Therapy	Non-randomized trial with pre- and post-test					2. HADS Hospital Anxiety and Depression Scale checklist (Zigmond & Snaith, 1983)
							3. WSAS Work and Social Adjustment Scale (Mundt, Marks, Shear, & Greist, 2002)
							4. Friendship Questionnaire ad hoc not validated
7. Wise, y otros, 2019	Modular Cognitive Behavioral Therapy (MBCT) in groups	Open rehearsal	16	60	7	16 y 20	1. ADOS (Autism Diagnostic Observation Schedule) (Lord, y otros, 2012)
							2. ADIS-5 (Anxiety Disorders Interview (Brown & Barlow, 2013)
							3. Hamilton Anxiety Scale (HAM-A) (Hamilton, 1959)
							4. WASI-II; Wechsler Abbreviated Scale of Intelligence-Second Edition (Wechsler, 2011)
							5. BAI (Beck & Steer, 1993)
							6. CGI-S (Clinical Global Impression of Severity (Guy, 1976)

1. Ehrenreich-May, J., Simpson, G., Stewart, L., Kennedy, S., Rowley, A., Beaumont, A., . . . Wood, J. (2020). Treatment of anxiety in older adolescents and young adults with autism spectrum disorders: A pilot study. *Bulletin of the Menninger Clinic*, 84 (2), 105-136. doi:https://doi.org/10.1521/bumc_2020_84_03.

2. Gaigg, S., Flaxman, P., McLaven, G., Shah, R., Bowler, D., Meyer, B., . . . South, M. (2020). Self-guided mindfulness and cognitive behavioural practices reduce anxiety in autistic adults: A pilot 8-month waitlist controlled trial of widely available online tools. autism, 24(4), 867-883. doi:https://doi.org/10.1177/1362361320909184.

3. Ordaz, D., Lewin, A., & Storch, E. (2018). Modular Cognitive-Behavioral Therapy for a Young Adult with Autism Spectrum Disorder, Comorbid Obsessive Compulsive Disroder and Social Anxiety Disorder. Clinical Case Studies, 17(3), 136-149. doi:https://doi.org/10.1177/1534650118770269.

4. Parr, J., Brice, S., Welsh, P., Ingham, B., Le Couteur, A., Evans, G., . . . Rodgers, J. (2020). Treating anxiety in autistic adults: study protocol for the Personalised Anxiety Treatment–Autism (PAT-A©) pilot randomised controlled feasibillity trial. *Trials*, 21, 265. doi:https://doi.org/10.1186/s13063-020-4161-2.

5. Sizoo, B., & Kuiper, E. (2017). Cognitive behavioural therapy and mindfulness based stressreduction may be equally effective in reducing anxiety anddepression in adults with autism spectrum disorders. *Research in Developmental Disabilities*, 64, 47-55. doi:https://doi.org/10.1016/j.ridd.2017.03.004.

6. Spain, D., Blainey, S., & Vaillancourt, K. (2017). Group cognitive behaviour therapy (CBT) for social interaction anxiety in adults with autism spectrum disorders (ASD). *Research in Autism Spectrum Disorders*, 41-42, 20-30. doi:https://doi.org/10.1016/j.rasd.2017.07.005.

7. Wise, J., Cepeda, S., Ordaz, D., McBride, N., Cavitt, M., Howle, F., . . . Storch, E. (2019). Open Trial of Modular Cognitive-Behavioral Therapy in the Treatment of Anxiety Among Late Adolescents with Autism Spectrum Disorder. *Child Psychiatry & Human Development*, 50, 27-34. doi:https://doi.org/10.1007/s10578-018-0817-5
